# Genome-wide identification of the PI-PLC gene family in *Setaria italica* and functional characterization of SiPLC1 in salt stress response

**DOI:** 10.3389/fpls.2025.1694096

**Published:** 2025-12-09

**Authors:** Dan Zhu, Xiaobing Hu, Feng Feng, Mengqi Tian, Yonghu Zhang, Ran Chai, Rui Wen, Jianhua Wei, Jiewei Zhang

**Affiliations:** 1 Yellow River conservancy Technical University, Kaifeng, China; 2 Inner Mongolia Academy of Agricultural & Animal Husbandry Sciences, Huhhot, China; 3 Beijing Key Laboratory of Agricultural Genetic Resources and Biotechnology, Beijing Key Laboratory of Crop Molecular Design and Intelligent Breeding, Beijing Academy of Agriculture and Forestry Sciences, Beijing, China

**Keywords:** foxtail millet, phosphoinositide-specific phospholipase C, SiPLC genes family, SiPLC1, salt stress

## Abstract

Foxtail millet (*Setaria italica*), a typical NADP-ME-type C4 crop, demonstrates superior light-use and water-use efficiencies compared to C3 species. Beyond its photosynthetic edge, it employs a range of stress-resilience mechanisms that optimize growth-defense trade-offs under drought, salinity, or nitrogen scarcity. The combination of high C4 photosynthetic efficiency with extensive stress tolerance is rare among cultivated species, positioning foxtail millet as an ideal model for studying the integration of yield and stress resilience. Phosphoinositide-specific Phospholipase C (PI-PLC) plays a crucial role in lipid- and Ca^2+^-dependent signaling pathways. In plants, it modulates responses to biotic and abiotic stresses, though the mechanisms remain partially understood. In this study, we identified five *PI-PLC*-encoding genes in foxtail millet, named *SiPLC1-SiPLC5*, and analyzed their systematic phylogeny, gene structure, protein characteristics, distribution of the chromosome, collinearity relationship, and *cis-*acting elements prediction at the promoter region. Phylogenetic analysis revealed that the members of the *SiPLCs* gene family were divided into three subgroups. Structural analysis that all of them have four conserved motifs and these motifs were evenly distributed. Notably, *SiPLC1* harbors an exceptionally large first intron and falls within subgroup II; its protein sequence is highly homologous to *AtPLC1* and *AtPLC3* of *Arabidopsis thaliana* (L.) Heynh. and to *OsPLC4* of *Oryza sativa* L. RT-qPCR indicated that *SiPLC1* is predominantly expressed in roots during early stem elongation and is significantly upregulated under salt stress. Overexpression of *SiPLC1* in *Arabidopsis* mitigated salt-induced damage, highlighting its critical role in salt-stress signal transduction in foxtail millet.

## 1 Introduction

Foxtail millet (*Setaria italica* (L.) P. Beauv) is a gramineous crop and one of the most important and ancient cereal crops cultivated in northern China. It is highly valued for its nutritional richness, strong climate adaptability, and low cultivation input, and is widely grown in many arid and semi-arid regions (Goron and Raizada, 2015). Foxtail millet exhibits broad adaptability and stable yield performance, enabling it to thrive under adverse weather conditions, with notable tolerance to high temperatures and drought stress (Yuan et al., 2021; Zhu et al., 2025). Foxtail millet possesses a stable root architecture, small leaf area, and thick cell walls, along with strong stress resistance and high water and nutrient use efficiency. It demonstrates resilience to multiple abiotic and biotic stresses (Pant et al., 2016; Kasinathan et al., 2024). Additionally, foxtail millet has the advantages of a small diploid genome (~430 Mb), a short life cycle, and C4 photosynthesis (Zhang et al., 2012). Compared to other major crops such as *Triticum aestivum* L. and *Zea mays* L., *S. italica* exhibits superior water use efficiency and requires less water. These characteristics make it an ideal model crop for studying fundamental biological features, including plant structure, physiology, and genomic evolution (Yang et al., 2020). Therefore, expanding the cultivation and research of foxtail millet contributes to safeguarding food security, mitigating global climate change, diversifying human dietary nutrition, advancing fodder and forage production, and providing a robust model system for fundamental studies in Poaceae biology.

Soil salinization is one of the most pressing environmental challenges worldwide. Elevated salt concentrations disrupt plant ionic and osmotic homeostasis, leading to physiological dysfunction, severe yield losses, and even total crop failure. With increasing groundwater overexploitation, excessive fertilizer use, and global soil salinization, salt stress is anticipated to become a significant constraint on foxtail millet productivity in the future. Elucidating the molecular mechanisms underlying salinity-alkalinity tolerance and mining the corresponding genetic resources are therefore essential for enhancing plant resilience, reclaiming saline-sodic soils, and restoring degraded ecosystems. Plants have evolved intricate molecular signaling networks to respond to salt stress, primarily involving abscisic acid (ABA)-dependent and ABA-independent pathways (Fujii et al., 2009; Nakashima and Yamaguchi-Shinozaki, 2013; Yoshida et al., 2014). In the ABA-dependent pathway, osmotic stress induced by salt triggers ABA synthesis and accumulation, which activates the SnRK2 protein kinase family. These kinases then regulate the downstream AREB/ABF and other bZIP transcription factors through phosphorylation, inducing the expression of typical stress response genes such as *RD29A* and *RD22* (Uno et al., 2000; Yamaguchi-Shinozaki and Shinozaki, 1994). This pathway also involves the upregulation of key ABA biosynthesis genes, such as *NCED3*, and the negative feedback regulation of PP2C-type phosphatases, including *ABI1* and *ABI2*, forming a dynamic regulatory loop (Bai et al., 2013; Woo et al., 2011). In the ABA-independent pathway, salt stress signals directly activate the MAPK cascade, leading to the expression of DREB/CBF transcription factors. DREB2A, for instance, governs the transcription of genes like *KIN1* and *COR15A* by binding to DRE/CRT *cis-*acting elements in their promoters (Kidokoro et al., 2022; Mizoi et al., 2012). Importantly, these pathways are not entirely independent; they interact at multiple levels. Some DREB-like transcription factors are indirectly influenced by ABA signals, while certain ABA components are directly triggered by osmotic stress. This interaction forms a synergistic regulatory network that enhances plants’ adaptability to salt stress (Zhu, 2016).

Phosphoinositide-specific phospholipase C (PI-PLC) is a key enzyme in the phosphoinositide signaling pathway, widely conserved across animals, plants, yeast, and bacteria (Suh et al., 2008; Roberts et al., 2018). In animal cells, PI-PLC hydrolyzes phosphatidylinositol 4,5-bisphosphate (PIP2) to generate the second messengers inositol 1,4,5-trisphosphate (IP3) and diacylglycerol (DAG). The water-soluble IP3 enters the cytoplasm, promoting Ca^2+^ influx from intracellular stores and modulating Ca^2+^-dependent enzymes and channels. The lipid-soluble DAG remains in the membrane, activating protein kinase C (PKC), which then regulates the activity of various enzymes, receptors, transporters, and cytoskeletal components through phosphorylation, thereby participating in diverse cellular processes (Boss and Im, 2012; Kadamur and Ross, 2013; Meijer and Munnik, 2003). In plants, the understanding of the PI-PLCs is based on animal research. Plant PI-PLCs generate IP_3_ or its derivatives, which release Ca^2+^ from intracellular stores, with Ca^2+^ playing a crucial role in regulating growth, development, and stress adaptation. DAG is rapidly converted to phosphatidic acid (PA), another second messenger. PIP2, IP3, DAG, and PA ald to various external stimuli and stresses (Laxalt et al., 2025; Melin et al., 1987; Nakamura et al., 2005; Rupwate and Rajasekharan, 2012). Most plant PI-PLC enzymes contain four typical motifs: EF, X, Y, and C2. Among them, the X and Y motifs are highly conserved and together form a TIM barrel fold, which serves as the core region essential for PI-PLC catalytic function (Hong et al., 2016). It is noteworthy, however, that not all active PI-PLCs possess complete X/Y motifs; for example, *Arabidopsis* AtPLC8 and AtPLC9 exhibit significant deletions in the Y motif compared to other family members (Pokotylo et al., 2014). The C2 motif is also conserved and is primarily involved in phospholipid recognition and enzyme activation, a process generally mediated by Ca²^+^ (Helling et al., 2006). The EF motif, located at the N-terminus of PI-PLCs, shows considerable sequence variation among different plant species and is less conserved. This motif is believed to play a role in subcellular localization and may enhance the affinity between the enzyme and its substrates (Kadamur and Ross, 2013; Munnik and Testerink, 2009; Pokotylo et al., 2014). Recent studies have expanded our understanding of plant PI-PLC functions. In the *A. thaliana*, the expression of AtPLC6, AtPLC7, and AtPLC8 is upregulated by auxin, cytokinin, and stress conditions such as salinity, drought, and cold (Tasma et al., 2008; Chua et al., 2001). AtPLC1 is rapidly induced by salt stress and its function is critical for salt tolerance (Hirayama et al., 1995). AtPLC3 plays a role in the plant’s response to high-temperature stress (Ren et al., 2017; Zheng et al., 2012). AtPLC2 regulates phosphatidic acid production by modulating salicylic and jasmonic acid levels, thereby enhancing resistance to endoplasmic reticulum respond drought stress (Kanehara et al., 2015). AtPLC9 is crucial for heat stress response, influencing intracellular Ca^2+^ and IP3 levels and regulating heat shock protein (HSP) gene expression. Knockout of AtPLC9 diminishes thermotolerance, while its overexpression increases it. A double knockout of AtPLC3 and AtPLC9 results in even lower thermotolerance than individual knockouts (Ren et al., 2017). AtPLC4 is involved in salt stress response regulation (Xia et al., 2017). In the *O. sativa*, OsPLC4 knockout mutants show reduced IP3 and cytosolic Ca^2+^, alongside suppressed Ca^2+^ sensors and stress-related genes, leading to lower osmotic stress tolerance. Overexpression of OsPLC4 improves growth and survival of *Arabidopsis* seedlings under high salt and drought conditions (Deng et al., 2019). OsPLC1 is also implicated in the regulation of salt stress response in rice (Li et al., 2017; Wang et al., 2023; Yu et al., 2022). OsPLC3 plays a crucial role in rice osmotic stress responses by maintaining ROS homeostasis (Yu et al., 2021). In the *Solanum lycopersicum* L., heat treatment markedly upregulates *SlPLC3* and *SlPLC6* expression (Vossen et al., 2010). While in maize, ZmPLC1 is associated with drought stress response (Wang et al., 2008), and *ZmPLC5* is upregulated under low-temperature stress (Dong et al., 2018). PetPLC1 influences *PIP2* and *actin* distribution, providing genetic evidence for PI-PLCs involvement in plant polar growth (Dowd et al., 2006). In potato, *StPLC1* transcript levels decrease under wounding and drought, whereas *StPLC2* levels sharply increase, indicating its role in stress signaling (Kopka et al., 1998). Increasing research highlights the involvement of *PI-PLC* genes in cell development and signal transduction pathways under various stresses. In crops like rice, corn, tobacco, potato, and tomato, *PI-PLC* genes expression is modulated by biotic and abiotic stresses. Enhancing specific *PI-PLC* genes expression can significantly bolster plant tolerance to stresses such as salinity and drought, making them promising targets for genetic engineering to develop stress-tolerant, high-yielding crops. However, the PI-PLC family members in foxtail millet are not fully identified, and their biological roles remain unclear. This study aims to systematically characterize the *PI-PLC* genes family in foxtail millet to elucidate its potential role in stress adaptation mechanisms, thereby providing important insights into the enhanced resilience of C4 plants. Comprehensive phylogenetic reconstruction and comparative genomic analysis systematically elucidated the evolutionary relationships between SiPLC members in foxtail millet and their functionally characterized orthologs in model plants such as *A. thaliana* and *O. sativa*. Based on high sequence homology with known salt-tolerance genes *AtPLC1* and *OsPLC4*, enrichment of stress-responsive *cis-*acting elements in its promoter region, and distinctive gene structural features, SiPLC1 was selected as a key candidate for in-depth functional characterization. Our findings establish a crucial foundation for understanding phosphoinositide-mediated signaling in C4 species and propose novel theoretical frameworks for improving stress tolerance in cereal crops through molecular breeding strategies.

## 2 Materials and methods

### 2.1 Plant materials

The foxtail millet cultivar Yugu 1 and the Arabidopsis genotype Col-0 were obtained from Beijing Crop Germplasm Resources Infrastructure and used in this study. 35-day-old foxtail millet seedlings cultivated in a greenhouse were exposed to diverse stress conditions, including high salinity (200 mM NaCl), high alkalinity (550 mM KOH), low temperature (4°C), and high temperature (42°C) treatments (Zhu et al., 2020), as well as drought stress (roots were washed, dried with absorbent paper, and placed on 3M filter paper) (Cai et al., 2014). Following a 6 hour treatment period, the seedlings underwent real-time quantitative PCR (RT-qPCR) analysis.

Transgenic Arabidopsis seeds were germinated on MS medium supplemented with 2.5% sucrose and 0.5% phytagel (pH 5.75) and cultivated vertically under illumination at 22°C for 5 days. Subsequently, seedlings displaying uniformity in shoot size and primary root length (approximately 0.8 cm) were transplanted onto MS media supplemented with either 50 mM, 100 mM, or 150 mM NaCl, all containing 2.5% sucrose and 0.5% phytagel. After a further 10 days of growth, images were captured, and measurements of fresh weight and primary root length were conducted.

### 2.2 Identification PI-PLCs in the foxtail millet genome

The complete genome, proteome, and gene structure annotation data for foxtail millet were retrieved from the Phytozome database (https://phytozome-next.jgi.doe.gov/info/Sitalica_v2_2). The basic sequences of the *PI-PLC* gene family members in *A. thaliana* were downloaded from TAIR version 9.0 (http://www.Arabidopsis.org/) to serve as query sequences. The complete genome sequence files of the rice *PI-PLC* gene family were obtained from the NCBI database (https://www.ncbi.nlm.nih.gov/). The Hidden Markov Model (HMM) (PF00179) for the *PI-PLC* gene family was downloaded from the Pfam database (http://www.pfam.org). The TBtools Protein Parameter Calc tool was then employed to determine the basic physical and chemical properties, including motif composition and molecular weight, of the identified *PI-PLC* gene family members.

### 2.3 Multiple sequence alignment and phylogenetic analysis

The PI-PLC protein sequences of *S. italica*, *O. sativa*, and *A. thaliana* were aligned using the Clustal W method in the MEGA7 software. Default parameters were employed during the alignment process, and a phylogenetic tree was constructed based on the conserved sequences. The Neighbor-Joining (N-J) algorithm of the genetic distance algorithm was used, and the Bootstrap value was set to 1000. The resulting phylogenetic tree was further modified and annotated using the Evolview online software (https://evolgenius.info//evolview-v2). Additionally, collinear gene pairs between *SiPLCs* and *OsPLCs*, *SiPLCs* and *AtPLCs* were identified using the MCScanX software (https://github.com/wyp1125/MCScanX), and the collinear relationships were visualized using the TBtools Multiple Synteny Plot tool (Bailey et al., 2009; Finn et al., 2016).

### 2.4 Chromosome location, gene structure, conserved motifs analysis of SiPLCs

The chromosomal distribution and structural characteristics of the *SiPLCs* gene family were investigated. The genomic coordinates of the *PI-PLC* genes were extracted from the foxtail millet genome annotation file using the Gene Location Visualization tool in TBtools software, and their chromosomal distribution was visualized. The conserved motifs within the *PI-PLC* gene family were identified using the MEME online program and the NCBI Batch CD-search tool, respectively. The gene structures were further visualized using the Gene Structure View (Advanced) tool in TBtools.

### 2.5 Analysis of *Cis-*acting elements in the promoter

The promoter regions of the *SiPLCs* gene family in foxtail millet were analyzed to identify *cis-*acting regulatory elements. The 2000 bp upstream sequences were extracted using TBtools and submitted to the PlantPAN3.0 platform for prediction of *cis*-acting elements. Further screening revealed the presence of hormone response and stress resistance elements. The distribution of these regulatory elements was visualized using the Basic Bioequivalence View tool in TBtools.

### 2.6 Systematic analysis of *SiPLC1* expression patterns and stress responses in the foxtail millet

To investigate the tissue-specific expression pattern of *SiPLC1* during the early shooting stage and its response to various abiotic stresses, the following experiments were conducted: 1) Tissue-Specific Expression Analysis: Young roots, stems, and leaves were harvested in the early shooting stag. Total RNA was extracted from each tissue and converted to cDNA through reverse transcription. Subsequently, RT-qPCR was performed using this cDNA to evaluate the expression of *SiPLC1* in different tissues, with the aim of identifying potential functional variations among plant organs. 2) Abiotic Stress Treatments and Expression Profiling: Plants were subjected to high salt, alkaline, drought, low temperature, and high temperature stresses. Following a 6-hour treatment period, total RNA was isolated from roots and stems for cDNA synthesis. RT-qPCR was carried out using the StepOnePlus™ Real-Time PCR System (Applied Biosystems) with three biological replicates (10 plants each). *SiActin* and *SiDIREF1α-2* served as the internal control, and all reactions were conducted following the SYBR Green kit protocol. The relative expression levels of *SiPLC1* were determined using the 2^–ΔΔCt^ method.

### 2.7 Acquisition and Verification of *Arabidopsis* Transformed with *SiPLC1*

The *SiPLC1* gene was cloned into the pCAMBIA1300 vector, which was maintained in our laboratory. The recombinant construct was transformed into Agrobacterium tumefaciens GV3101 competent cells, which were then used to transform *Arabidopsis* plants via the Agrobacterium-mediated method, generating the T0 generation of *Arabidopsis* overexpressing *SiPLC1.* The T0 plants were selected on hygromycin-containing medium to identify positive transformants. Homozygous lines were subsequently isolated based on Mendelian inheritance principles for experimental use.

The expression of the SiPLC1-GFP fusion protein in transgenic plants was verified through immunoblot analysis. Total proteins were extracted from leaves using an extraction buffer, as previously described (Li et al., 2016). A monoclonal Anti-GFP antibody (1:10,000) was used to detect the target fusion protein, and a horseradish peroxidase-conjugated goat Anti-mouse antibody (1:10,000) served as the secondary antibody. The chemiluminescent signal generated by the enzyme-labeled secondary antibody upon binding to the target protein was captured on X-ray film, producing a visible image that corresponds to the presence and localization of the SiPLC1-GFP fusion protein.

### 2.8 Statistical analysis

The statistical analysis in this study was conducted as follows: Data are presented as the mean ± SEM from independent experiments. For comparisons between two groups, the *t*-test was used. The significance thresholds were set at *P* < 0.05 (*) and *P* < 0.01 (**). For comparisons across multiple groups, a one-way analysis of variance (ANOVA) was employed, followed by Tukey’s *post-hoc* test. The significance threshold was set at *P* < 0.05.

### 2.9 Image acquisition and processing via confocal and wide-field microscopy

Images of the fluorescent materials were acquired using a Zeiss LSM 710 confocal microscope. The collected images were subsequently processed with ZEN software; Morphological observations were performed using an Olympus BX53 upright optical microscope; High-magnification images were acquired with a motorized Olympus BX53 research microscope equipped with a DP80 high-resolution digital camera.

## 3 Results

### 3.1 Identification and bioinformatics analysis of the *PI-PLC*s in foxtail millet

**Figure 1:**
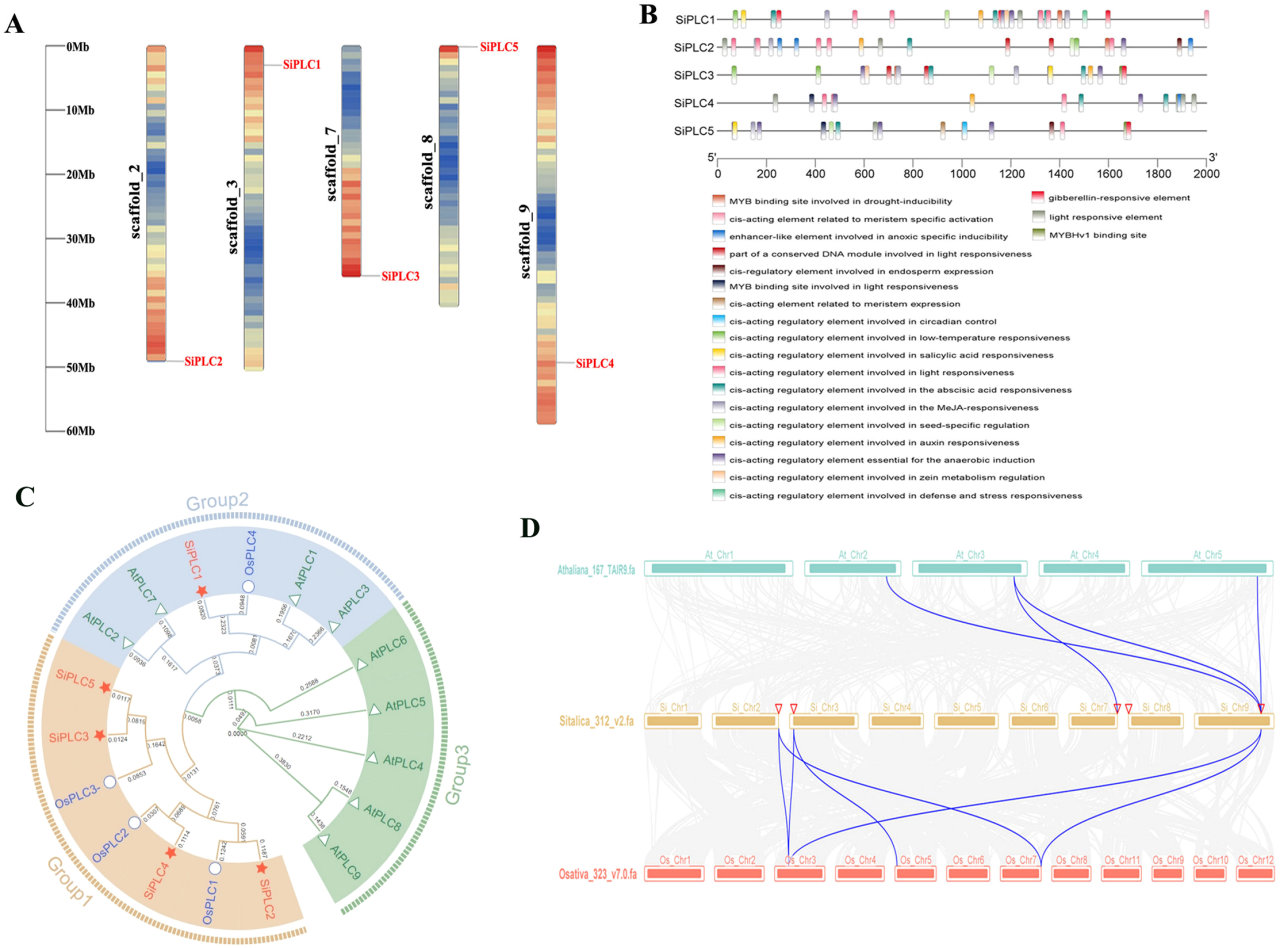
Sequence and structure analysis of the *SiPLC* genes family. **(A)** The distribution of five *PI-PLC* genes across 5 chromosomes in foxtail millet. Depicts the distribution of five PI-PLC genes across the five chromosomes of foxtail millet. **(B)** Depicts the *cis-*acting element analysis of the *SiPLC* genes promoter. Various *cis-*acting elements with distinct regulatory functions are delineated within the 2000 bp promoter region of the *SiPLC* genes, with their respective locations indicated. The 2000 bp promoter sequence is denoted by a black line, while colored rectangles symbolize different categories of *cis-*acting elements. **(C)** Phylogenetic relationships of PI-PLCs in *S. italica* (Si), *A. thaliana* (At) and *O. sativa* (Os). The phylogenetic tree was generated using the neighbor-joining method with MEGA 7 software. PI-PLCs in foxtail millet were marked with different labels and diverse subgroups of PI-PLCs were highlighted by different colors. **(D)** Result of colinearity analysis for *PI-PLC* genes in three species: *S. italica*, *A*. *thaliana*, and *O. sativa*. Collinear relationships between *PI-PLC* genes of different species are highlighted by blue lines.

Following the identification of 5 unique *SiPLC* genes (*SiPLC1* to *SiPLC5*) in the Yugu1 genome, a comprehensive physicochemical analysis was conducted. The coding sequence lengths of these genes ranged from 1770 to 1908 base pairs, encoding proteins of 589 to 635 amino acids, molecular weight ranging from 66221.38 to 71104.88 Da. The predicted isoelectric points (pI) of the SiPLC proteins varied from 5.81 to 6.32, with the majority exhibiting a acidic nature (pI < 7) due to their enrichment in acidic amino acids. Furthermore, the instability indices ranged from 40.13 to 45.31, the aliphatic indices fell between 70.15 and 76.55, and all SiPLC proteins were predicted to possess hydrophilic properties (**Supplementary Table 1**). To explore the evolutionary relationships between foxtail millet, the dicot model plant *A. thaliana*, and model monocot plant *O. sativa*, we compared the *PI-PLC* gene families of these species. A phylogenetic tree was constructed using PI-PLC proteins sequences from *A. thaliana*, *O. sativa*, and *S. italica*. The tree divided all PI-PLC members into three groups. Subgroup I had the largest members, including SiPLC2-SiPLC5, OsPLC1-OsPLC3; subgroup II had the 6 members, including SiPLC1, AtPLC1-AtPLC3, AtPLC7 and OsPLC4; subgroup III had AtPLC4-AtPLC6, AtPLC8, AtPLC9, with 5 members. Generally, having homologous groups showed monophyletic relationships, with foxtail millet PI-PLC members clustering more closely with rice PI-PLC proteins than with those from *A. thaliana*. The phylogenetic analysis reveals a closer evolutionary relationship between foxtail millet and rice, consistent with their taxonomic placement within the Poaceae family (**Figure 1C**). Notably, SiPLC1 clusters phylogenetically within the same subclade as three functionally characterized PI-PLC proteins: AtPLC1 and AtPLC3 from *A. thaliana*; OsPLC4 from *O. sativa*, both of which have been documented to play roles in salt stress response. Sequence alignment reveals that SiPLC1 shares 47.49% similarity with AtPLC1 and 79.97% with OsPLC4 (**Supplementary Figure 1**). This high degree of phylogenetic conservation and sequence similarity strongly suggests that SiPLC1 may serve as a functional ortholog of these known salt-responsive PI-PLCs, likely operating through analogous biochemical pathways and regulatory mechanisms during salt stress adaptation in foxtail millet. To reveal the chromosomal distribution of *SiPLC* genes, we mapped the physical locations of each *SiPLC* genes onto 5 chromosomes of foxtail millet. The results showed that the five members of this gene family (*SiPLC1-SiPLC5*) are dispersed across chromosomes on scaffold_2, scaffold_3, scaffold_7, scaffold_9, and scaffold_8 (**Figure 1A**).To further explore the function of the *SiPLC* genes, we searched the promoter region 2000 bp upstream of the transcription start site of the *SiPLC* genes in the Plant Promoter database (PlantCARE). The promoter regions of the *SiPLC* genes family exhibit an abundance of stress-responsive regulatory elements. SiPLC1 harbors the highest number of such elements. These promoters are enriched with regulatory motifs that confer responsiveness to various stimuli, including low temperature, drought, salicylic acid, abscisic acid, as well as plant hormones such as methyl jasmonate, auxin, and gibberellin. Furthermore, regulatory elements associated with light responsiveness, seed development, meristem activity, and endosperm expression were also identified (**Figure 1B**). These findings suggest the *SiPLC* genes family plays a crucial role in plant stress adaptation, hormone signaling, and growth regulation, highlighting its potential in plant stress resistance and genetic improvement research. In addition, to understand the collinearity of *PI-PLC* genes among different species,we performed a collinearity analysis of *PI-PLC* genes in three representative species: *S. italica*, *A. thaliana*, and *O. sativa*. The findings indicated that more *PI-PLC* genes in foxtail millet than in Arabidopsis exhibited multiple homologous counterparts with irregular chromosomal distributions in rice, indicating a closer evolutionary association between the two Poaceae species (**Figure 1D**).

**Figure 2:**
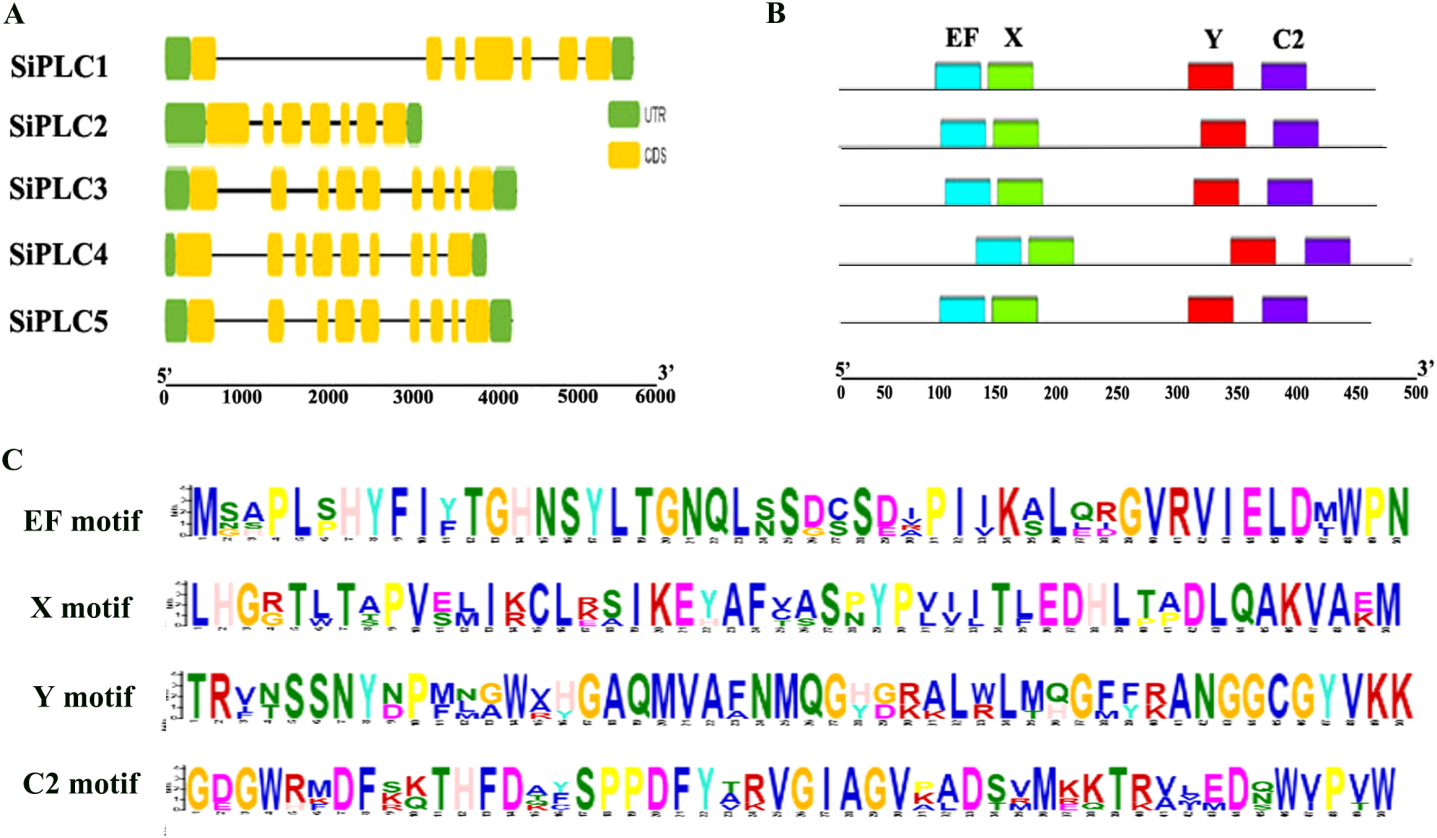
Conserved motifs and gene structural features of SiPLCs. **(A)** Gene structure distribution of the *SiPLC* genes family in foxtail millet. Green bars represent UTRs; yellow bars represent exons; and black lines represent introns. **(B)** Distribution of conserved motifs in SiPLC proteins. The consensus-conserved motifs of SiPLCs proteins were identified in the MEME suite web server. Blue bars represent “EF” motifs; green bars represent “X” motifs; red bars represent “Y” motifs; Purple bars represent “C2” motifs. **(C)** Sequence alignment of representative SiPLCs depicts the four conserved motifs: EF, X, Y, and C2.

To gain a clearer and more intuitive understanding of the evolutionary relationships among SiPLC members, we visualized their gene structures and motifs. Analysis of gene structure revealed variations in the length of DNA sequences, as well as intron and exon counts among different individuals within the same group. Among *SiPLCs* member, *SiPLC1* and *SiPLC2* have the same number of introns and exons, they both have 7 exons and 6 introns. In contrast, *SiPLC3-SiPLC5* contains more exons and introns, they all have 9 exons and 8 introns. It is noteworthy that the *SiPLC1* contains a particularly large first intron, a feature absent from the other genes examined (**Figure 2A**). A conserved motif analysis showed that five genes all have four conserved motifs: the EF motif, the PI-PLC catalytic motif (comprising elements X and Y), and the C2 motif (**Figure 2B**), and these motifs were evenly distributed, and SiPLC proteins within the same subgroup shared similar motif compositions and arrangements (**Figure 2C**).

### 3.2 Tissue specific expression analysis of the *SiPLC1* in the foxtail millet

**Figure 3:**
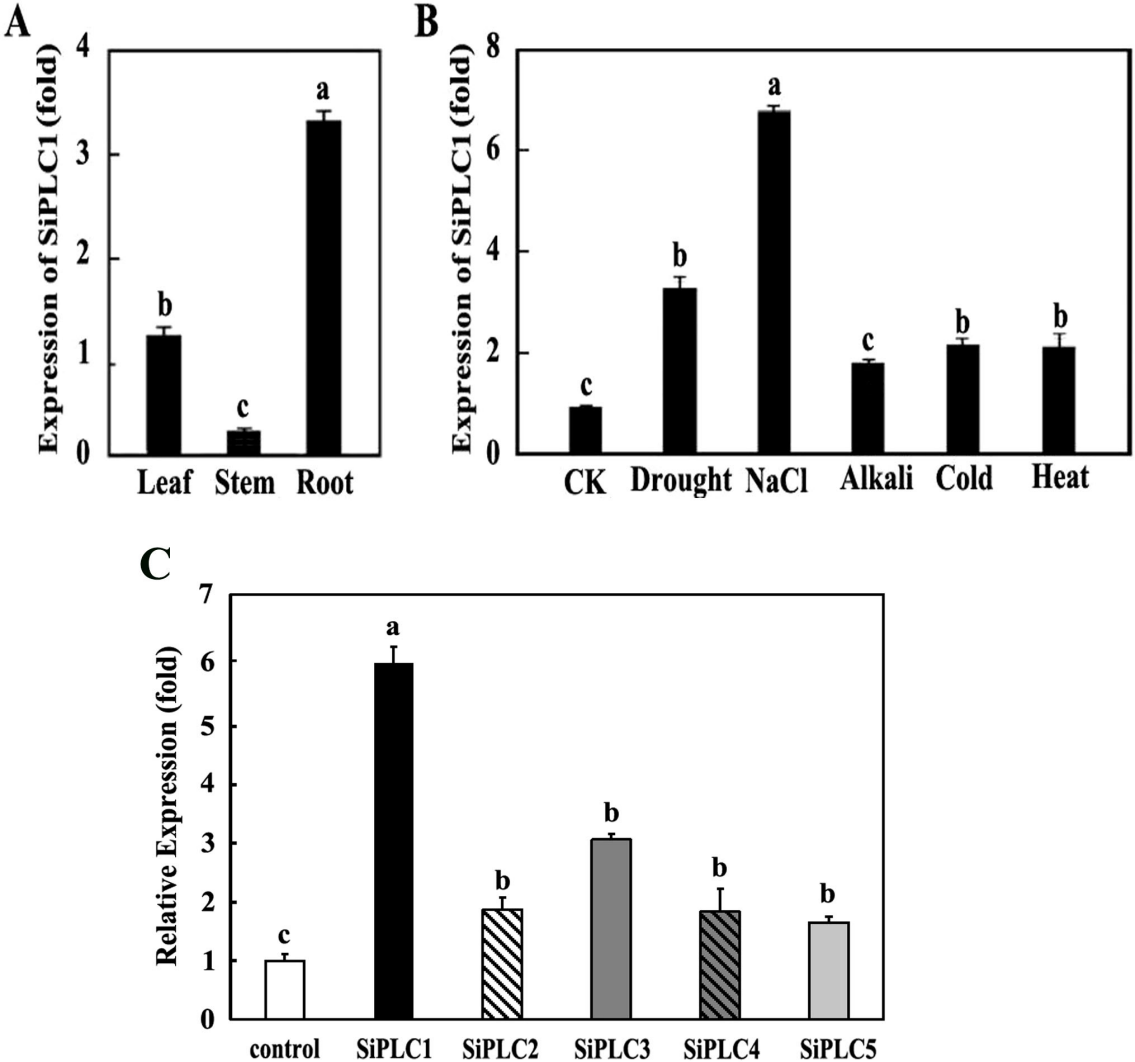
*SiPLC1* expression patterns under different treatments in foxtail millet **(A)***SiPLC1* gene expression in various tissue of 35-day-old foxtail millet seedlings, including leaf, root, stem. **(B)***SiPLC1* gene expression under drought, 200 mM NaCl, 550mM KOH, 4°C and 42°C. The *SiActin* and *SiDIREF1α-2* were used as an internal reference. In response to the various stress treatments, Root were specifically selected for analysis. All assays were repeated three times, and all values were means of three replicates with 10 plants. **(C)** The varied expression patterns of the PI-*PLC* genes family in foxtail millet under salt stress. Transcript levels of all *SiPI-PLC* genes in roots were assessed using RNA-seq following NaCl treatment. *SiActin* and *SiDIREF1α-2* were used as internal reference genes. Data are presented as mean ± SEM (*n* = 3). Group differences were analyzed by one-way ANOVA with Tukey’s *post-hoc* test, with *P* < 0.05. Different letters indicate a statistically significant difference. If two bars have no letters in common, it means the difference between them is statistically significant (P < 0.05). The same letter indicates no statistically significant difference. If two bars share at least one common letter, it means there is no statistically significant difference between them.

Understanding a protein’s subcellular localization and tissue-specific expression is essential for elucidating its biological role. To explore the spatial expression of the *SiPLC1*, we conducted RT-qPCR on root, stem, and leaf tissues from foxtail millet. Our findings indicate that *SiPLC1* is expressed in all tissues examined, with notable differences in transcript levels (**Figure 3A**). Expression was highest in roots, followed by leaves, while stems showed minimal expression-approximately 15% of that in roots. This expression pattern suggests *SiPLC1* may be crucial for root development or physiological processes at this stage, given its elevated transcription in roots. In contrast, its low expression in stems suggests a limited role in shoot development during this phase.

To assess the *SiPLC1* gene’s response to stress, foxtail millet was exposed to different stressors, and RT-qPCR was employed to quantify *SiPLC1* transcriptional changes. Results indicated that stress conditions led to an upregulation of *SiPLC1* expression in roots relative to the control. High salinity elicited the most pronounced upregulation, approximately 7-fold compared to the control, followed by drought, which induced a 3.2-fold increase. High temperature and low temperature had similar induction effects, while alkali stress resulted in the least upregulation, about 1.5-fold that of the control (**Figure 3B**). In addition to this, we assessed the salt responsiveness of the SiPLCs family through expression profiling under salt stress. *SiPLC1* showed the most rapid and significant induction, with an 6-fold increase within 6 hours. Other SiPLC members exhibited weaker and delayed responses, with maximum induction levels between 1.5 to 3 folds. This early and strong transcriptional activation of *SiPLC1* is characteristic of a primary responder in stress signaling pathways (**Figure 3C**).

### 3.3 The SiPLC1 enhances plant salt stress resistance

To elucidate SiPLC1’s role in plant stress responses, we generated SiPLC1::GFP transgenic *Arabidopsis* via Agrobacterium-mediated transformation. Homozygous T2 lines were selected through hygromycin resistance and confirmed by genomic PCR. Two independent overexpression lines (*OE-2* and *OE-5*) with stable transgene inheritance were chosen for detailed analysis. Transcriptional profiling showed *SiPLC1* expression in *OE-2* and *OE-5* lines was upregulated approximately 3.3-fold and 3-fold, respectively, compared to wild-type plants (WT) (**Supplementary Figure 2A**). Western blot analysis of total protein extracts using Anti-GFP antibodies identified a band of approximately 72 kDa, matching the predicted molecular weight of the SiPLC1-GFP fusion protein in transgenic plants (**Supplementary Figure 2B**). These results confirm the successful expression and proper translation of the *SiPLC1* transgene, establishing a foundation for further functional studies of SiPLC1 in plant stress responses.

**Figure 4:**
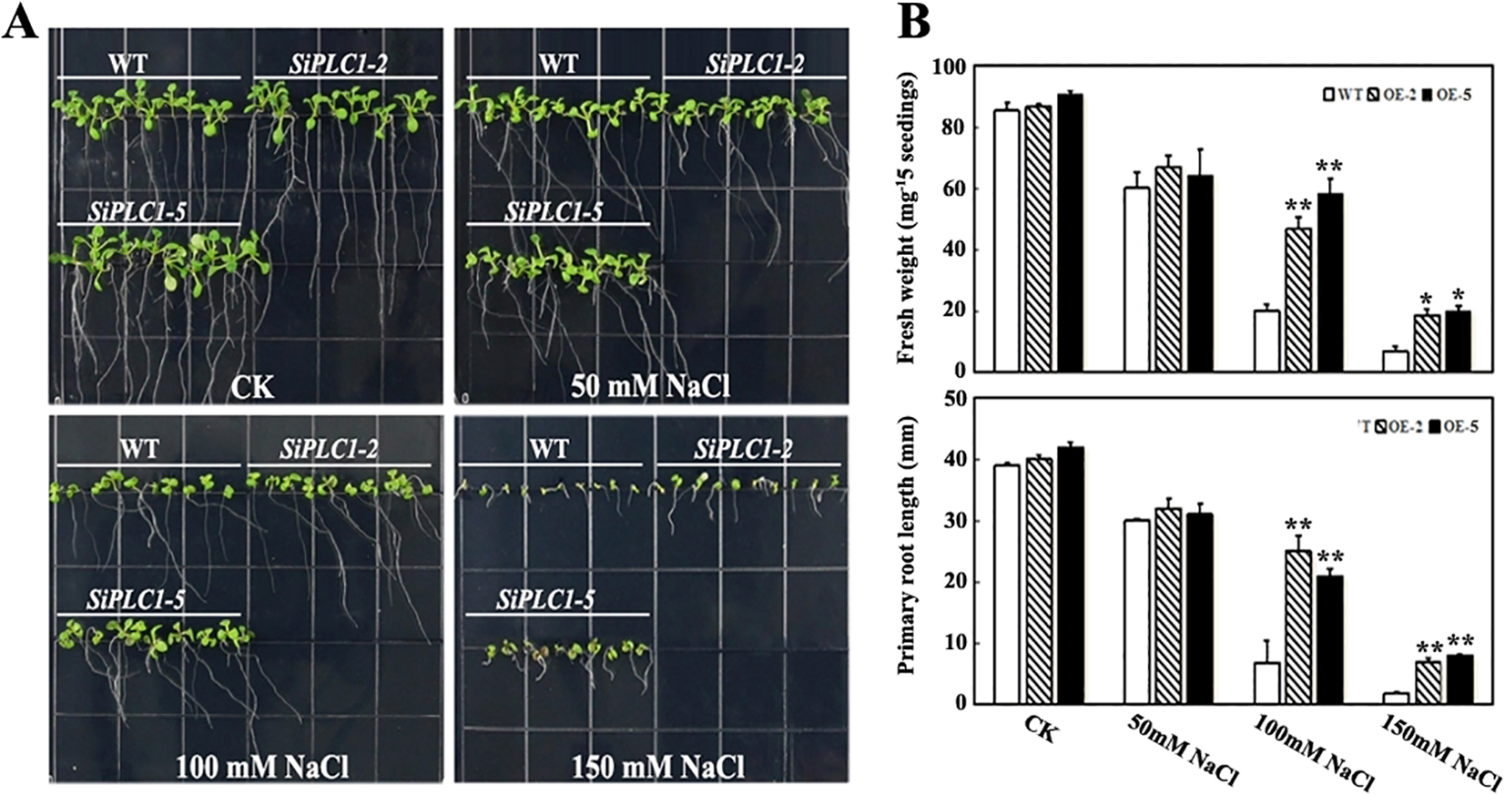
Overexpression of the *SiPLC1* gene enhances the salt stress resistance of the plant. **(A)** Analysis of salt sensitivity. 5-day-old WT, *OE-2* and *OE-5* transgenic seedlings grown on MS medium were transferred to MS medium with 50 mM, 100 mM, 150 mM NaCl. Photographs were taken 10 d after transfer. **(B)** Analysis of the fresh weight and primary root length of the seedlings in **(A)**. All assays were repeated three times, and all values were means of three replicates with 20 plants. *T-test* was used for differential analysis, Asterisk (*) indicates a difference at *P*<0.05 level, Asterisks (**) each column indicate a significant difference at *P*<0.01 level.

To elucidate SiPLC1’s role in seedling salt stress response, we examined the growth phenotypes of *SiPLC1* transgenic seedlings under varying salt concentrations. 5-day-old seedlings were transferred to Murashige and Skoog (MS) medium with 50 mM, 100 mM, and 150 mM NaCl and grown for 10 additional days. As depicted in figures, without NaCl, transgenic and control plants showed no growth differences, whereas upon NaCl exposure, all plants demonstrated salt stress sensitivity, with reduced aerial parts and shorter primary roots. Compared to WT and *GFP* transgenic *Arabidopsis*, *SiPLC1* transgenic *Arabidopsis* exhibited enhanced salt tolerance, manifesting as larger aerial parts and longer primary roots (**Supplementary Figure 3**; **Figure 4A**). Measurements of aerial fresh weight and primary root length revealed that, with increasing NaCl concentrations, the average fresh weight of *SiPLC1* transgenic lines exceeded WT by 9.2%, 66.8%, and 49.4%, and root length was 7.1%, 82.9%, and 116.4% longer, respectively (**Figure 4B**). Notably, WT exhibited substantial inhibition of lateral root growth under salt stress, whereas *SiPLC1* overexpressing lines preserved normal lateral root development (**Supplementary Figure 4**). Furthermore, subcellular localization analysis demonstrated that salt stress significantly increased the accumulation of SiPLC1 protein in root cells, with specific localization to the plasma membrane and nucleus (**Supplementary Figure 5**). These findings suggest that *SiPLC1* may enhance plant salt avoidance capacity by preserving normal root growth and through its specific subcellular localization, thereby improving overall salt tolerance.

**Figure 5:**
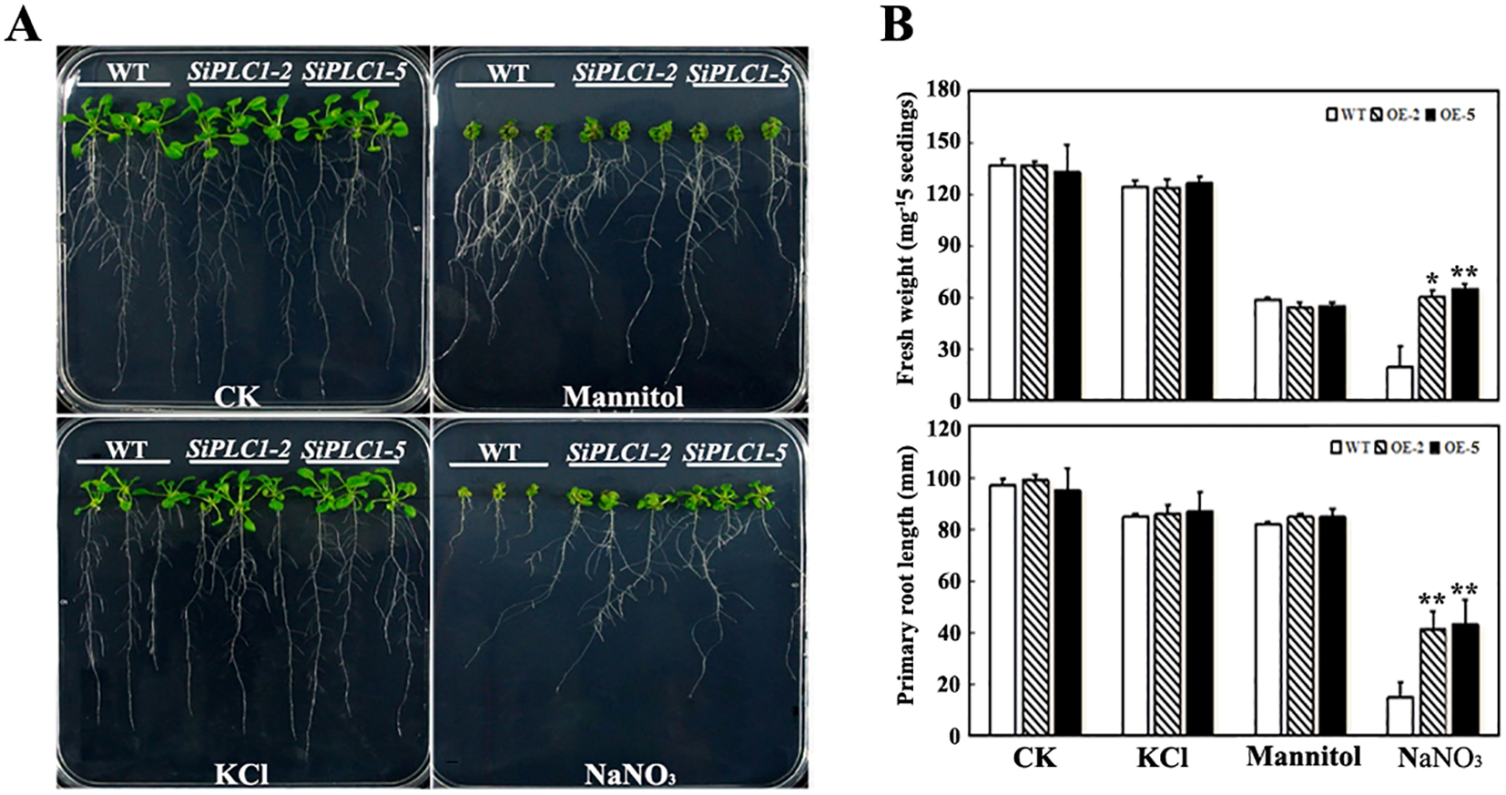
The salt tolerance of *SiPLC1* transgenic Arabidopsis plants is specific to sodium ion (Na^+^) stress. **(A)** Analysis of the resistance of the *SiPLC1* transgenic *Arabidopsis* to NaCl, KCl, NaNO_3_; and mannitol. All seedlings grown on MS medium were transferred to Ms medium with 100mM NaCl, 100 mM NaNO_3_, 100 m KCl and 187 mM mannitol. Photographs were taken 10 d after transfer. **(B)** Analysis of the fresh weight and primary root length of the seedlings in **(A)**. All assays were repeated three times, and all values were means of three replicates with 20 plants. *T-test* was used for differential analysis, Asterisk (*) indicates a difference at *P*<0.05 level, Asterisks (**) each column indicate a significant difference at *P*<0.01 level.

To isolate the effects of chloride ions and osmotic pressure from salt stress, we assessed the sensitivity of *SiPLC1* transgenic *Arabidopsis* to NaNO_3_, KCl, and mannitol. As depicted in **Figure 5**, there were no notable differences in growth phenotype, fresh weight, or primary root length between *SiPLC1* transgenic *Arabidopsis* seedlings and WT on MS medium with KCl or mannitol. However, on MS medium with NaNO_3_, *SiPLC1* transgenic *Arabidopsis* displayed reduced sensitivity to Na^+^, with fresh weight and primary root length increasing by 19% and 16%, respectively, compared to WT. These findings suggest that the reduced salt stress sensitivity in *SiPLC1* transgenic seedlings is specifically due to Na^+^, not chloride ions or osmotic pressure.

### 3.4 *SiPLC1* regulates the transcription of salt-responsive genes

**Figure 6:**
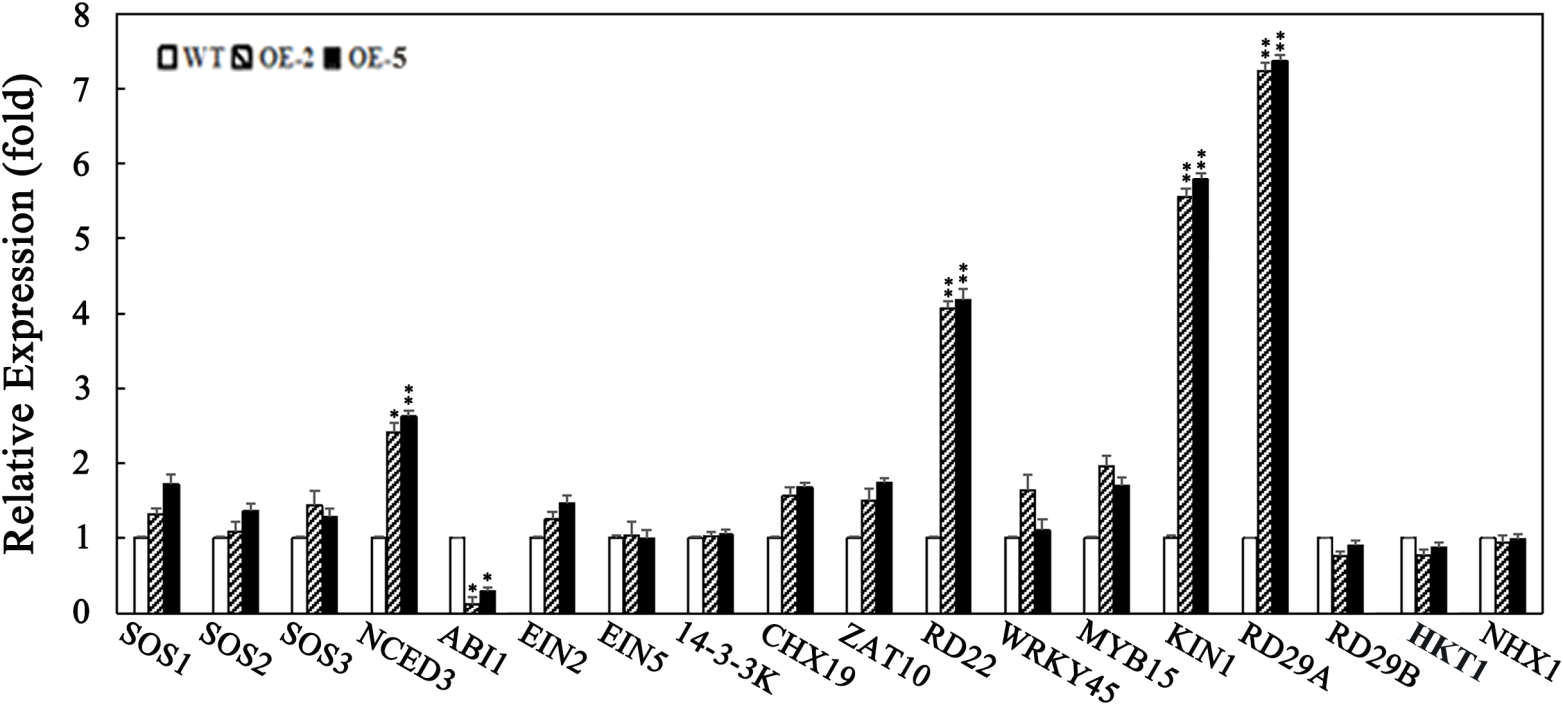
*SiPLC1* regulates the transcription of salt-responsive genes. Transcription detection of salt-responsive genes in WT, *OE-2*, and *OE-5* transgenic plants. 5-day-old seedlings grown on MS medium were transferred to MS medium with 100 mM NaCl for an additional 12h. Data are presented as mean ± SEM from three independent experiments. *T-test* was used for differential analysis, Asterisk (*) indicates a difference at *P*<0.05 level, Asterisks (**) each column indicate a significant difference at *P*<0.01 level.

Plant salt tolerance, a complex quantitative trait, is governed by the interplay of multiple genes involved in ion accumulation and exclusion, redox reactions, and osmolyte biosynthesis (Arif et al., 2020; Liu et al., 2019; Mishra and Tanna, 2017; Saad et al., 2010; Yu et al., 2014; Zhu, 2002). Salt stress influences the expression of numerous plant genes, with two primary gene groups responding at the transcriptional level (Bray et al., 2000; Shinozaki et al., 2003; Thomashow, 1999). The first group produces proteins that protect cells from dehydration, including enzymes for osmotic protectant synthesis such as late embryogenesis abundant (LEA) proteins, osmotic, chaperones, sugar and proline transporters, detoxification enzymes, and various proteases (Shinozaki and Yamaguchi-Shinozaki, 2007). The second group is involved in regulating gene expression and signal transduction (Kaur and Gupta, 2005), comprising transcription factors, protein kinases, calcium-dependent protein kinases (CDPKs), receptor-like kinases, and histidine kinases (Shinozaki and Yamaguchi-Shinozaki, 2007; Xiong et al., 2002; Zhu, 2002). Transcription factors bind to upstream promoter regions of genes, modulating transcription and playing a critical role in gene expression regulation under salt stress. Factors like MYB, NAC, bZIP, and WRKY act as key regulators in the gene regulatory network responding to salt stress (Arif et al., 2020; Chinnusamy et al., 2006; Liu et al., 2019). These genes are primarily involved in damage limitation or repair mechanisms (Zhu, 2001, 2003). To investigate SiPLC1’s role in salt stress regulation, we used quantitative PCR to identify stress-responsive genes in *SiPLC1* transgenic seedlings under salt stress. We analyzed genes known to respond to salt stress and found that SiPLC1 regulated the transcript levels of *SOS1, SOS2, SOS3, EIN2, EIN5, 14-3-3K, KIN1, CHX19, ZAT10, WRKY45, MYB15, HKT1, NHX1, NCED3, ABI1, RD22, RD29A, and RD29B* (**Figure 6**). Under normal conditions, stress-responsive gene expression was similar in *SiPLC1* transgenic *Arabidopsis* seedlings and WT. However, upon salt treatment, all tested genes were induced, with *SiPLC1* transgenic plants showing enhanced induction of *KIN1, NCED3, RD29A, RD22*, and reduced induction of *ABI1* compared to WT. These findings indicate that *SiPLC1* modulates the transcription of salt-responsive genes, highlighting its role in fine-tuning stress adaptation mechanisms.

## 4 Discussion

PI-PLC is integral to the plant phosphoinositide signaling pathway, playing crucial roles in abiotic stress response, cytoskeletal dynamics, and C4 crop carbon and nitrogen metabolism, among other physiological processes (Li et al., 2015; Takahashi et al., 2001; Zhang et al., 2015). Advances in genomics and bioinformatics have enabled the systematic identification of the *PI-PLC* genes family in *A. thaliana*, *O. sativa*, and other model plants (Singh et al., 2013). However, its genomic characteristics and biological functions in foxtail millet, a C4 crop noted for its stress tolerance, require further investigation. Comparative genomics reveals that the *PI-PLC* genes family comprises 9 members in *A. thaliana*, 4 in *O. sativa*, and 5 in *S. italica*. This variation highlights the evolutionary diversity of the family, suggesting that different members may undergo functional differentiation, contributing to tissue-specific expression and environmental signal response. Structural analysis revealed that the *PI-PLC* genes feature four characteristic motifs: EF-hand, X, Y, and C2 (**Figure 2B**). This indicates a high degree of sequence and structural conservation within the PI-PLC family in foxtail millet. Promoter *cis-*acting elements analysis showed that all SiPLC members possess multiple stress response elements, with SiPLC1 having the most, suggesting its potential central role in stress regulation (**Figure 1B**). Phylogenetic analysis indicated that SiPLC1 is most closely related to OsPLC4, AtPLC1 and AtPLC3 (**Figure 1C**). This suggests that SiPLC1 may function similarly to AtPLC1 and OsPLC4. Notably, gene structure analysis revealed that SiPLC1 has a significantly enlarged first intron, which is not only much longer than its other introns but also larger than the first introns of other family members (**Figure 2A**). Introns are non-coding sequences that interrupt protein-coding exons in eukaryotic genomes. During pre-mRNA splicing, introns are precisely excised while exons are ligated to form continuous coding sequences. The resulting mature mRNA is then translated into functional proteins. In contrast, the excised intronic RNA fragments are rapidly degraded by cellular surveillance mechanisms and do not participate in protein synthesis (Chan and Pek, 2019). Consequently, intronic RNAs have historically been considered mere transcriptional byproducts with no biological significance, leading to their relative neglect in molecular research. Since their discovery, introns have been recognized as a defining characteristic of eukaryotic genomes, exhibiting remarkable variation in both abundance and length across species. Despite their prevalence, the potential biological functions of introns remain poorly understood, representing a significant gap in our knowledge of genome biology. Echnological advances in the 20 century first uncovered the regulatory potential of introns in metazoans. In 1983, Banerji demonstrated that discovered that in mice, the enhancers of the heavy chain genes of immunoglobulins are located in their introns, and the activity of the enhancers in the introns is cell-specific. The regulatory function of this intron is independent of its position and orientation relative to the promoter (Banerji et al., 1983). In plants, the earliest functional evidence came from maize: deletion of the first intron of Alcohol dehydrogenase-1 (*Adh1*) decreased reporter expression 50 to 100 fold (Callis et al., 1987). Subsequently, in the studies of *Arabidopsis*, rice and maize, plant biologists discovered many introns with regulatory functions by removing or inserting intron fragments into other genes through restriction endonucleases. Such as *Arabidopsis* Phytochrome a signal transduction 1 genes (*PAT1*) (Rose and Last, 1997), Agamous (*AG*) (Hong et al., 2003); Rice genes Tubulin Alpha Chain 1 (*TubA1*) (Jeon et al., 2000); Maize gene Ubiquitin (*Ubi*) (Christensen et al., 1992), and so on. The regulatory role of these introns is manifested as intron-mediated enhancement of gene expression. This study revealed that the SiPLC1 contains a remarkably extended first intron, a structural feature not observed in other family members or homologous genes. This discovery provides novel insights into the regulatory functions of introns. Further investigations employing CRISPR-based editing, 3C technology, and reporter gene assays will be conducted to elucidate the underlying molecular mechanisms.

Our study revealed that the *SiPLC1* gene responds to multiple abiotic stresses, with particularly pronounced induction under high-salinity conditions (**Figure 3B**). To further investigate its biological function in salt stress response, we generated *Arabidopsis* transgenic lines overexpressing *SiPLC1.* The transgenic lines were rigorously validated through both RT-qPCR analysis confirming *SiPLC1* overexpression and immunoblot detection of the GFP-tagged protein (**Supplementary Figure 2**). Subsequently, confirmed positive transgenic plants were subjected to salt stress treatments to assess their phenotypic responses. Our research demonstrated that the *SiPLC1* transgenic *Arabidopsis* plants exhibited significantly enhanced salt resistance compared to WT (**Figure 4**). To explore the mechanism by which SiPLC1 regulates salt stress response, we analyzed the transcript levels of salt-responsive genes in *SiPLC1* overexpression plants. Under salt stress, all tested genes exhibited increased expression, yet key components of canonical ion homeostasis pathways, such as *SOS* and *NHX*, did not show significant transcriptional upregulation. This finding implies that SiPLC1-mediated salt tolerance likely operates independently of the classical SOS signaling module. Compared to WT, *SiPLC1* transgenic lines exhibited enhanced expression of *KIN1, RD29A, RD22*, and *NCED3*, concomitant with reduced induction of *ABI1*. Several studies have also reported that the *AtPLC1* regulates the expression of *KIN1, RD29A* and *RD22* in response to ABA (Sanchez and Chua, 2001). In the biosynthetic pathway of ABA, NCED3 are key regulatory enzymes (Woo et al., 2011). ABI1 is a type of protein phosphatase 2C (PP2C), which acts as a negative regulator in the ABA signaling transduction pathway (Bai et al., 2013). Furthermore, systematic analysis of the *cis-*acting elements in both the promoter of *SiPLC1* identified multiple ABA-responsive motifs, providing molecular support for its potential role in ABA-mediated stress signaling. Based on these findings, we hypothesize that SiPLC1 participates in ABA-dependent salt stress response. SiPLC1 may regulate ABA-related gene expression by modulating transcription factor activity, potentially through altering PI-PLC activity, thereby influencing the expression of genes involved in ABA-dependent salt stress adaptation. The precise regulatory pathways involved require further investigation.

In this investigation, Phosphoinositide-specific phospholipase C gene family were successfully identified from foxtail millet and designated as *SiPLC1-SiPLC5*. Sequence analysis demonstrated five genes encoded proteins exhibit a domain organization typical of plant PI-PLCs. Phylogenetic analysis revealed that SiPLC1 shares high sequence homology with AtPLC1, AtPLC3 and OsPLC4, clustering together in subgroup II. Subsequent examinations demonstrated that the *SiPLC1* gene is predominantly expressed in the roots of foxtail millet, where it is involved in regulating responses to salt stress. These findings provide a critical theoretical and empirical foundation for further elucidating the biological functions of SiPLC1 protein in the growth, development, and stress responses of foxtail millet.

## Data Availability

The original contributions presented in the study are included in the article/**Supplementary Material**. Further inquiries can be directed to the corresponding author.

## Supplementary material

Supplementary Figure 1 Multiple alignment of the SiPLCs and PI-PLCs proteins from other species.

Supplementary Figure 2 Molecular Characterization of *SiPLC1* Transgenic Plants. **(A)** RT-qPCR analysis of the *SiPLC1* transgenic Arabidopsis plants (*OE-1-OE-5*) and WT. Data are presented as mean ± SEM (*n* = 3). Group differences were analyzed by one-way ANOVA with Tukey’s *post-hoc* test, with *P* < 0.05. **(B)** Protein detection of *SiPLC1* overexpression seedlings and WT. *AtUBQ5* and *AtPP2A* were used as internal reference genes.

Supplementary Figure 3 Phenotypic comparison of SiPLC1::GFP and GFP-only transgenic *Arabidopsis* under salt stress. **(A)** Phenotype of SiPLC1::GFP transgenic *Arabidopsis* under salt stress. **(B)** Phenotype of GFP transgenic *Arabidopsis* under salt stress. The SiPLC1::GFP overexpression lines show enhanced sensitivity to salt stress compared to the GFP-only controls. This contrast establishes a specific role for SiPLC1 in mediating salt hypersensitivity, independent of the GFP tag.

Supplementary Figure 4 *SiPLC1* overexpression enhances root hair development under salt stress. **(A)** Root phenotypic of *SiPLC1* overexpression plants after salt treatment. 7-day-old seedlings precultured on normal 1/2 MS medium were transferred to medium with or without 150 mM NaCl and grown vertically for 10 days. Compared with WT, *SiPLC1* overexpression lines (*OE-2*, *OE-5*) exhibited a more developed root hair system under salt stress. **(B)** Magnified images of root hairs after salt treatment. Scale bar=1 cm. **(C)** Ratio of branched root hairs. Results show that the ratio of branched root hairs in overexpression lines was significantly higher than in WT. Experiments were independently repeated three times with consistent results. Data are presented as mean ± SEM (*n* = 20). Group differences were analyzed by one-way ANOVA with Tukey’s *post-hoc* test, with *P* < 0.05.

Supplementary Figure 5 Salt stress significantly enhanced both the accumulation and specific localization of SiPLC1 protein at the plasma membrane and nucleus in *Arabidopsis* root cells.
