# Supplementary figures and images for: Genome-wide identification of the PI-PLC gene family in Setaria italica and functional characterization of SiPLC1 in salt stress response
Source: Front Plant Sci. 2025 Dec 9;16:1694096. doi: 10.3389/fpls.2025.1694096 (PMC12722992; doi:10.3389/fpls.2025.1694096)

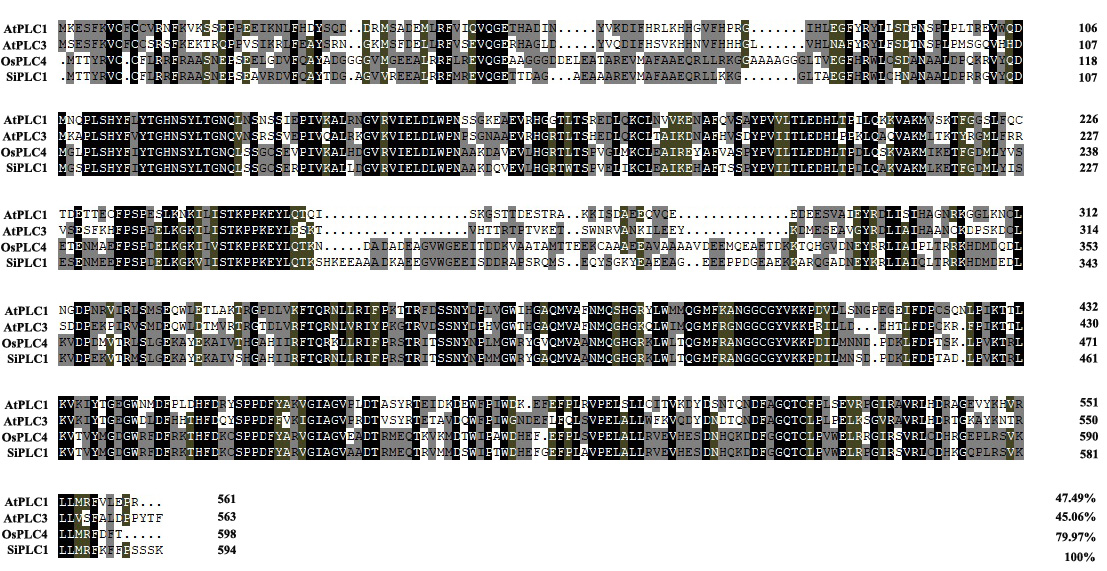

Supplement: Supplementary Figure 1 — Multiple alignment of the SiPLCs and PI-PLCs proteins from other species. [file Image1.jpeg]

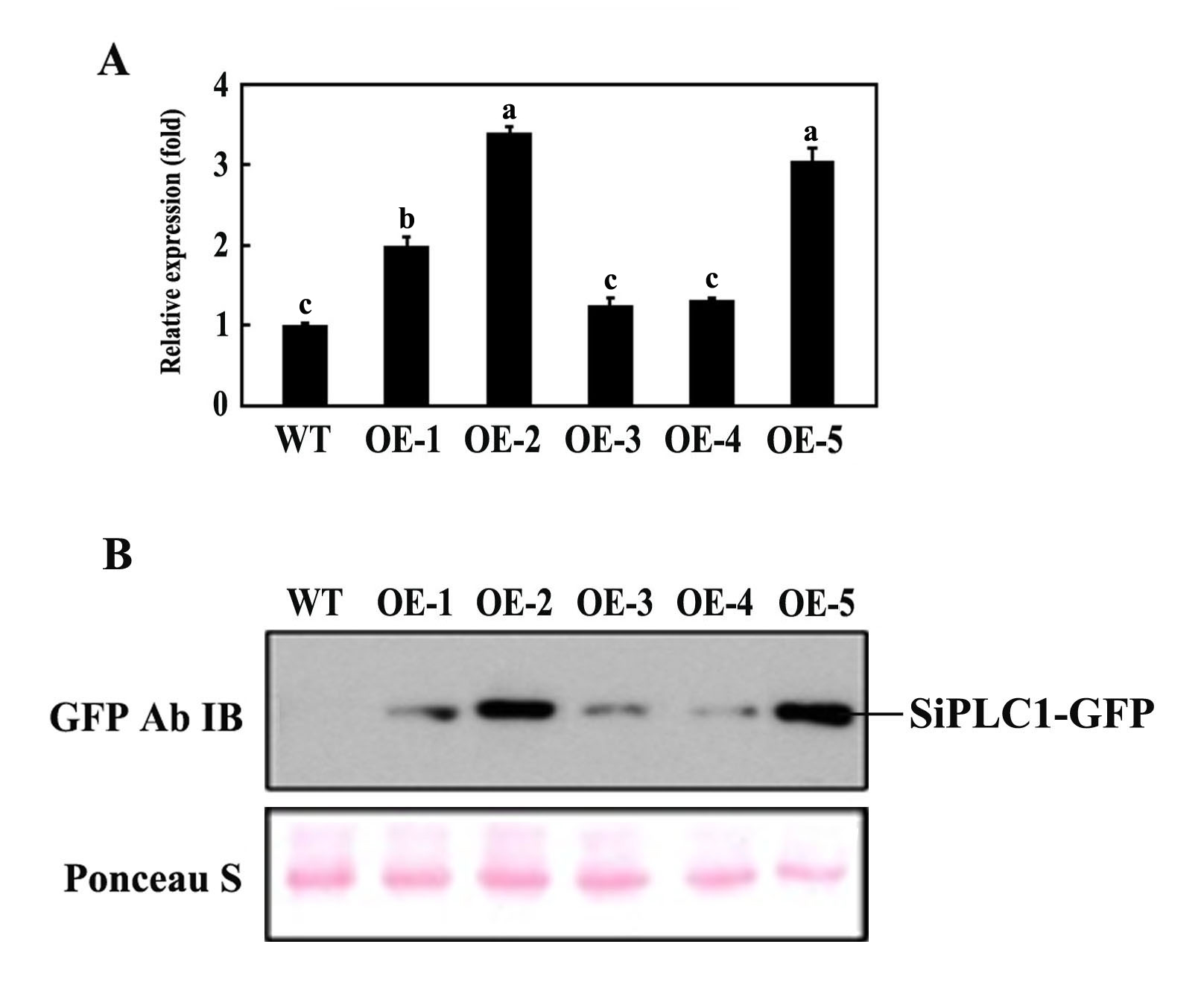

Supplement: Supplementary Figure 2 — Molecular Characterization of SiPLC1 Transgenic Plants. (A) RT-qPCR analysis of the SiPLC1 transgenic Arabidopsis plants (OE-1-OE-5) and WT. Data are presented as mean ± SEM (n = 3). Group differences were analyzed by one-way ANOVA with Tukey’s post-hoc test, with P < 0.05. (B) Protein detection of SiPLC1 overexpression seedlings and WT. AtUBQ5 and AtPP2A were used as internal reference genes. [file Image2.jpeg]

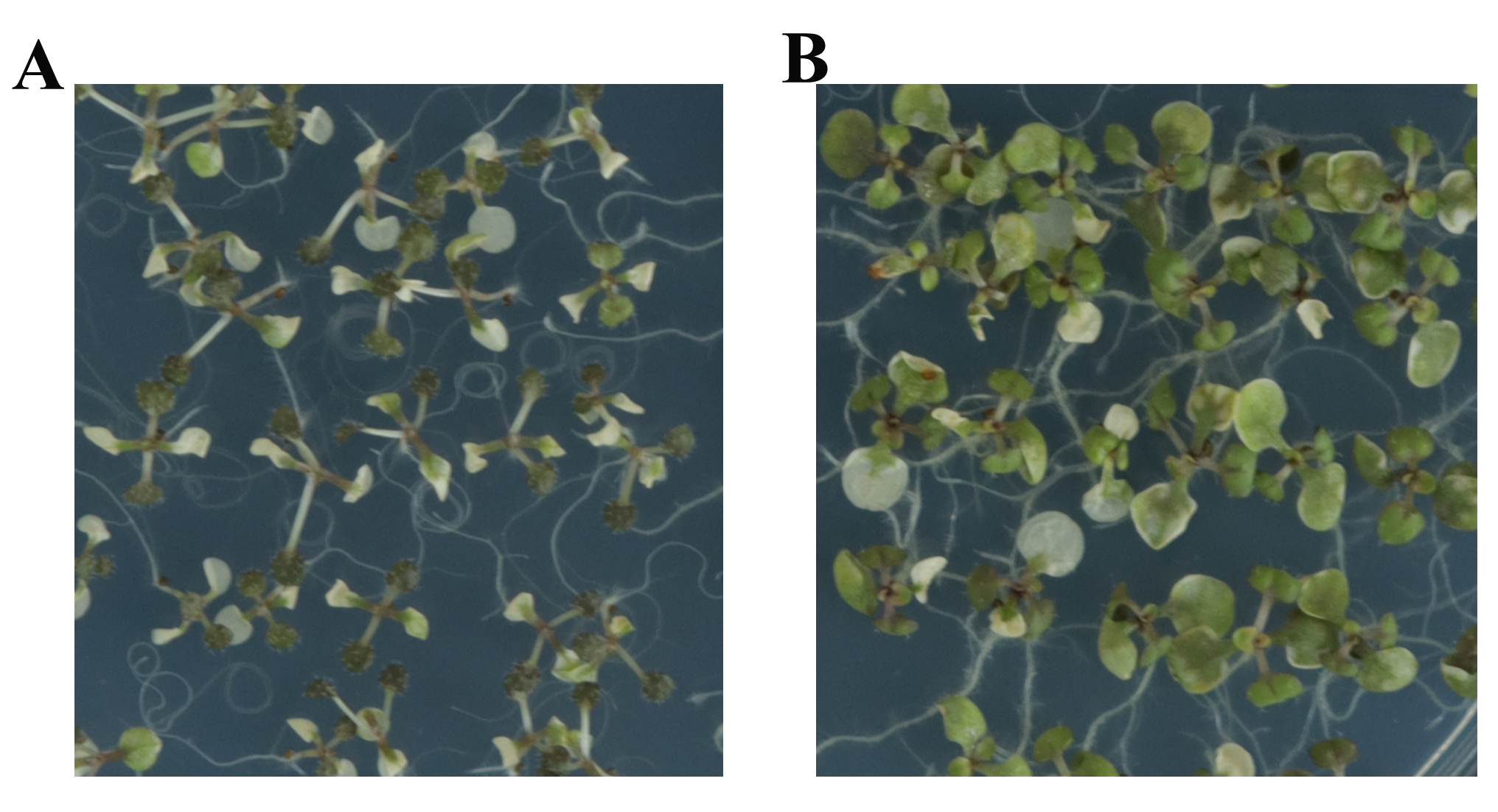

Supplement: Supplementary Figure 3 — Phenotypic comparison of SiPLC1::GFP and GFP-only transgenic Arabidopsis under salt stress. (A) Phenotype of SiPLC1::GFP transgenic Arabidopsis under salt stress. (B) Phenotype of GFP transgenic Arabidopsis under salt stress. The SiPLC1::GFP overexpression lines show enhanced sensitivity to salt stress compared to the GFP-only controls. This contrast establishes a specific role for SiPLC1 in mediating salt hypersensitivity, independent of the GFP tag. [file Image3.jpeg]

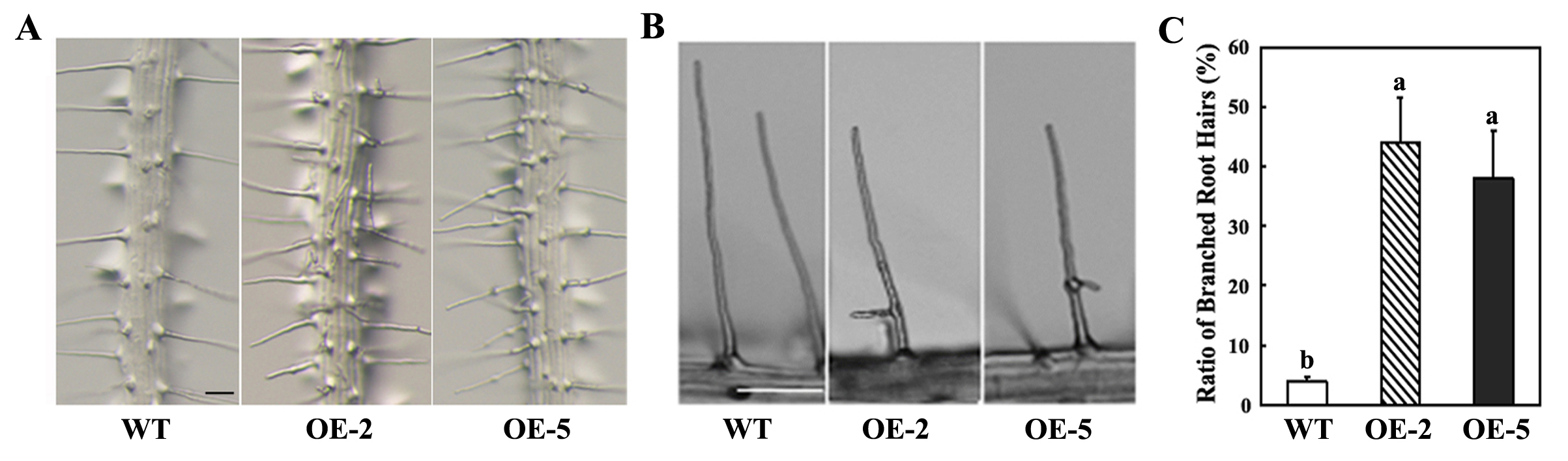

Supplement: Supplementary Figure 4 — SiPLC1 overexpression enhances root hair development under salt stress. (A) Root phenotypic of SiPLC1 overexpression plants after salt treatment. 7-day-old seedlings precultured on normal 1/2 MS medium were transferred to medium with or without 150 mM NaCl and grown vertically for 10 days. Compared with WT, SiPLC1 overexpression lines (OE-2, OE-5) exhibited a more developed root hair system under salt stress. (B) Magnified images of root hairs after salt treatment. Scale bar=1 cm. (C) Ratio of branched root hairs. Results show that the ratio of branched root hairs in overexpression lines was significantly higher than in WT. Experiments were independently repeated three times with consistent results. Data are presented as mean ± SEM (n = 20). Group differences were analyzed by one-way ANOVA with Tukey’s post-hoc test, with P < 0.05. [file Image4.jpeg]

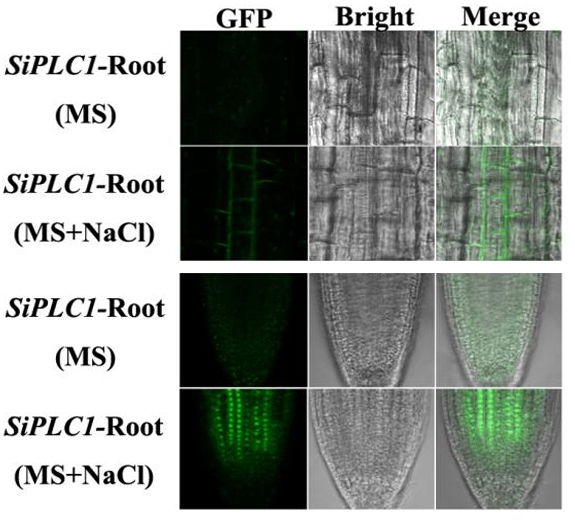

Supplement: Supplementary Figure 5 — Salt stress significantly enhanced both the accumulation and specific localization of SiPLC1 protein at the plasma membrane and nucleus in Arabidopsis root cells. [file Image5.jpeg]
